# Supplementary material for: The interactome of intact mitochondria by cross-linking mass spectrometry provides evidence for coexisting respiratory supercomplexes
Source: Mol Cell Proteomics. 2017 Dec 8;17(2):216–32. doi: 10.1074/mcp.RA117.000470 (PMC5795388; doi:10.1074/mcp.RA117.000470)
Supplement: Supplemental Data [file supp_RA117.000470_133922_0_supp_23978_fzffwf.zip › spectra_annotation/mito_DR_spectra_annotation/337-1-13-1-4-1.pdf]

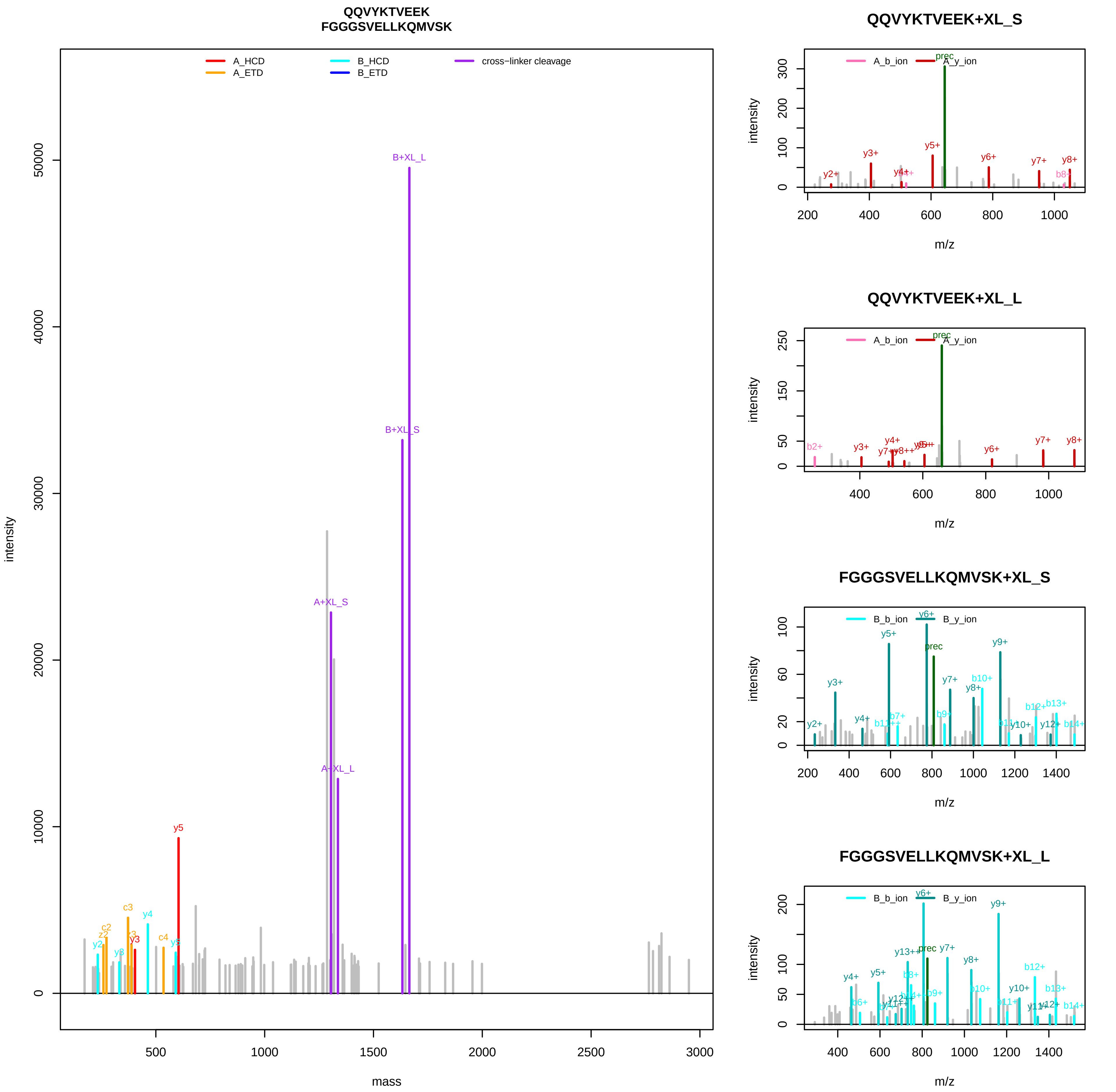

QQVYKTVEEK
FGGGSVELLKQMVSK
A_HCD
A_ETD
B_HCD
B_ETD
cross−linker cleavage
B+XL_L
B+XL_S
A+XL_S
A+XL_L
y5
c3
y4
c2
z2
y2
y3
c4
intensity
mass
0
10000
20000
30000
40000
50000
500
1000
1500
2000
2500
3000
QQVYKTVEEK+XL_S
A_b_ion
A_y_ion
prec
y2+
y3+
y4+
y5+
y6+
y7+
y8+
b8+
intensity
m/z
QQVYKTVEEK+XL_L
b2+
y3+
y4+
y6+
y7+
y8+
FGGGSVELLKQMVSK+XL_S
B_b_ion
B_y_ion
y2+
y3+
y4+
y5+
y6+
y7+
y8+
y9+
y10+
b7+
b9+
b10+
b12+
b13+
b14+
FGGGSVELLKQMVSK+XL_L
y4+
y5+
y6+
y7+
y8+
y9+
y10+
y13++
b6+
b8+
b9+
b10+
b12+
b13+
b14+
